# Response of tomatoes primed by mycorrhizal colonization to virulent and avirulent bacterial pathogens

**DOI:** 10.1038/s41598-022-08395-7

**Published:** 2022-03-18

**Authors:** Moeka Fujita, Miyuki Kusajima, Masatomo Fukagawa, Yasuko Okumura, Masami Nakajima, Kohki Akiyama, Tadao Asami, Koichi Yoneyama, Hisaharu Kato, Hideo Nakashita

**Affiliations:** 1 Department of Bioscience and Biotechnology, Fukui Prefectural University, Eiheiji, Japan; 2 Graduate School of Agricultural and Life Sciences, The University of Tokyo, Tokyo, Japan; 3 Faculty of Agriculture, Ibaraki University, Ami, Japan; 4 Graduate School of Life and Environmental Sciences, Osaka Prefecture University, Sakai, Japan; 5 Center for Bioscience Research and Education, Utsunomiya University, Utsunomiya, Japan

**Keywords:** Microbe, Biotic, Plant signalling

## Abstract

Most plants interact with arbuscular mycorrhizal fungi, which enhance disease resistance in the host plant. Because the effects of resistance against bacterial pathogens are poorly understood, we investigated the effects of mycorrhizal colonization on virulent and avirulent pathogens using phytopathological and molecular biology techniques. Tomato plants colonized by *Gigaspora margarita* acquired resistance not only against the fungal pathogen, *Botrytis cinerea,* but also against a virulent bacterial pathogen, *Pseudomonas syringae* pv. *tomato* DC3000 (*Pst*). In *G. margarita*-colonized tomato, salicylic acid (SA)- and jasmonic acid (JA)-related defense genes were expressed more rapidly and strongly compared to those in the control plants when challenged by *Pst*, indicating that the plant immunity system was primed by mycorrhizal colonization. Gene expression analysis indicated that primed tomato plants responded to the avirulent pathogen, *Pseudomonas syringae* pv. *oryzae,* more rapidly and strongly compared to the control plant, where the effect on the JA-mediated signals was stronger than in the case with *Pst*. We found that the resistance induced by mycorrhizal colonization was effective against both fungal and bacterial pathogens including virulent and avirulent pathogens. Moreover, the activation of both SA- and JA-mediated signaling pathways can be enhanced in the primed plant by mycorrhizal colonization.

## Introduction

Plants have several types of self-defense mechanisms against pathogens. Systemically induced disease resistance, activated by various types of stimuli, protects the plant for long periods from a broad range of attackers. Systemically induced defense plays an important role in allowing plants to survive even under harsh conditions in the environment. Some interactions between plants and pathogens activate these resistance mechanisms through phytohormone-governed signaling pathways. Salicylic acid (SA)-mediated defense response is effective against biotrophic pathogens, whereas jasmonic acid (JA)-mediated defense signaling is important for the resistance against necrotrophic pathogens^1,2^. Systemic acquired resistance (SAR), induced by the SA-mediated signaling pathway, is accompanied by the expression of defense-related genes, such as pathogenesis-related (PR) genes^3^. SAR is a relatively strong defense against subsequent pathogenic attacks; therefore, it has been used in the field by exploiting plant activators that induce SAR. Among these chemicals, a derivative of probenazole, 1,2-benzisothiazol-3(2*H*)-one1,1-dioxide (BIT) and benzo(1,2,3)thiadiazole-7-carbothioic acid *S*-methyl ester (BTH) are capable of activating upstream and downstream of SA biosynthesis, respectively, in the SAR signaling pathway^4,5^, which have been used for investigation of disease resistance mechanisms.

In addition to host–pathogen interactions, interactions with nonpathogenic or symbiotic microorganisms also activate plant immune systems. The rhizobacteria *Pseudomonas fluorescens* WCS417r^6,7^, *Bradyrhizobium* sp. ORS278^8^, and *Pseudomonas aeruginosa* 7NSK2^9^; the endophytic bacterium *Azospirillum* sp. B510^10,11^; and the arbuscular mycorrhizal fungi, *Rhizophagus irregularis* (formally *Glomus intraradices*)^12,13^ and *Funneliformis mosseae* (formerly *Glomus mosseae*)^14,15^, are among those that have been reported to induce disease resistance in host plants^16,17^.

Previous studies on the priming of immune system of plants by arbuscular mycorrhizal fungi, called mycorrhiza-induced resistance (MIR), indicate that MIR is effective against fungal^12,14,18–20^, bacterial^21^, and viral pathogens^22,23^ and, furthermore, against leaf-chewing caterpillars^24^ and aphids^25,26^. Although primed plants are able to respond more rapidly and strongly to pathogenic infection to protect themselves, no or weak expression of the major defense-related genes through SA- or JA-mediated signaling pathways were observed before pathogenic infection. Since these analyses have been performed only for MIR induced by *R. irregularis* and *F. mosseae,* analysis of MIR by other arbuscular mycorrhizal fungi will provide further insights into the mechanism of MIR.

Another arbuscular mycorrhizal fungus *Gigaspora margarita* Becker & Hall (*G. margarita*) can colonize tomato plants, however its effect on plant immunity has not been investigated. Because gathering large spores of *G. margarita* (260–480 µm in diameter)^27^ enable uniformity of colonization level among plants in experiments, this strain is suitable for characterization of plant immune systems in the mycorrhizal plants. Recent studies have revealed that effect of *G. margarita* colonization on plants growth is dependent on spore-associated bacteria interacting with spore surface and endobacteria existing in the fungal cytoplasm^28,29^.

Previous reports suggest that MIR is effective against necrotrophic pathogens but not against biotrophs such as foliar bacterial pathogens^30^. Furthermore, it has been reported that tobacco plants colonized with *R. irregularis* displayed reduced SA-mediated defense against infection with tobacco mosaic virus^31^. On the other hand, *Medicago truncatula* colonized with *R. irregularis* exhibited enhanced resistance against the virulent bacterial pathogen, *Xanthomonas campestris* pv. *Alfalfae*^21^. The question has been raised as to whether MIR is distinctly effective against biotrophic bacteria; however, only limited information is available on MIR against foliar bacterial pathogens. To better understand the effects of MIR on bacterial pathogens, we investigated the effects of MIR induced by *Gigaspora margarita* colonization on virulent and avirulent bacterial pathogens in tomato (*Solanum lycopersicum* L. cv. *Momotaro*) and the related priming effects on defense signaling. This is the first study to elucidate the priming responses against bacterial pathogens in MIR. The data presented here demonstrate the potentiality of MIR for controlling various diseases.

## Results

### Mycorrhiza induces disease resistance in tomato

**Figure 1:**
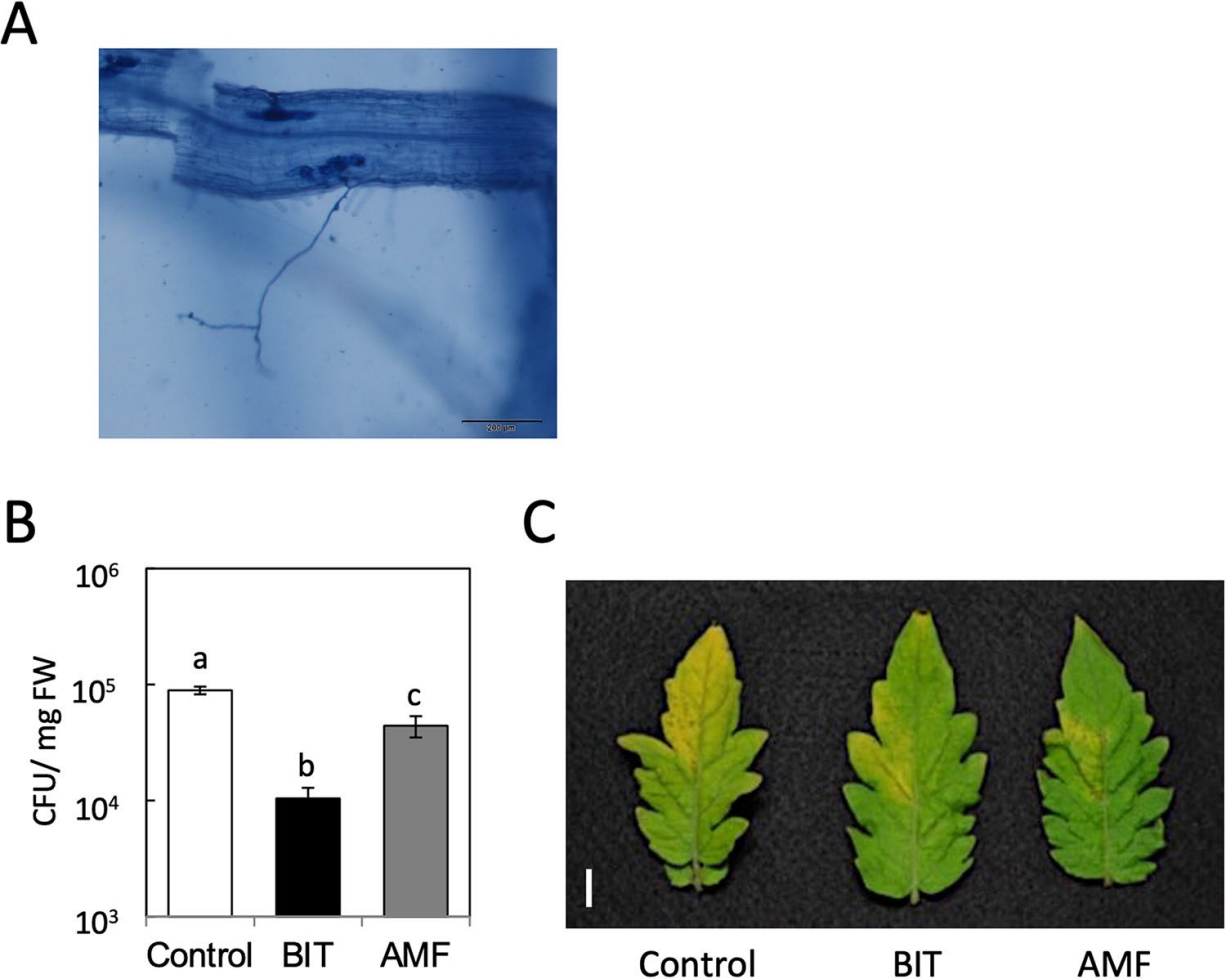
(**A**) Colonization of tomato root with *G. margarita*. Tomato plants treated with *G. margarita* (25 spores/pot) by soil drenching. Roots were washed and strained with trypan blue 6 days after the inoculation with *G. margarita.* Scale bar, 200 µm. (**B**) Induction of resistance against tomato leaf speck disease by *G. margarita*. Plants were treated with *G. margarita* (25 spores/pot) (AMF) 14 days prior to challenge inoculation with *Pst* (1 × 10^3^ CFU/ml). SAR was induced by treatment with BIT (5 mg/pot) by soil-drenching method 5 days prior to challenge inoculation. The growth of *Pst* in tomato leaflet was evaluated 2 days after the inoculation. Each experiment was done with more than 4 plants. Values are shown as the means ± SE (n = 8) of a single experiment. Different letters indicate statistically significant differences between treatments (one-way ANOVA with Tukey’s post-test, *p* < 0.05). The experiment was repeated three times with similar results. (**C**) Photograph of representative disease symptoms taken 5 days after inoculation with *Pst*. Scale bar, 10 mm.

To verify root-colonization by *G. margarita*, 14 and 28 days after inoculation (with 25 spores per plant), tomato roots were cleared and stained with trypan blue for microscopic evaluation of infection (Fig. 1A). The results revealed that the *G. margarita* colonization rate was only 1.5% at 14 days but increased to 10–15% at 28 days after inoculation.

Because *G. margarita*-colonized tomato plants (14 days after *G. margarita* inoculation) exhibited enhanced resistance against the necrotrophic fungal pathogen *Botrytis cinerea* (Supplementary Fig. S1)*,* we assessed the resistance against bacterial speck caused by *Pseudomonas syringae* pv. *tomato* DC3000 (*Pst*) in these plants. Resistance was determined by measuring bacterial growth in leaf tissues 2 days post challenge inoculation with *Pst*. As a positive control, SAR was induced with the SAR activator BIT. Treating tomato plants with BIT reduced bacterial growth compared to that in the water-treated control plants (Fig. 1B). The bacterial growth in the leaves of *G. margarita*-inoculated plants was less than half of that in the leaves of the water-treated control plants. At 5 days after inoculation, the inoculated leaves in the water-treated control exhibited a yellowish area spreading from the infected region widely in the leaflet (Fig. 1C). The disease severity in BIT-treated or *G. margarita-*inoculated plants was significantly reduced compared to that in the water-treated control (Fig. 1C). These results indicated that the symptoms of tomato bacterial speck were consistent with the in-planta growth of *Pst*. Antimicrobial activity against *Pst* was not detected in the extract of leaves of *G. margarita*-colonized plants (Supplementary Fig. S2). Thus, the resistance of *G. margarita*-colonized tomato against bacterial pathogens was caused by the activation of the plant immunity system.

To examine the involvement of spore-associated bacteria and endobacteria in disease resistance in *G. margarita*-colonized plants, spores were crushed using a pestle with a small amount of fine sea sand powder and then treated to the tomato root. The challenge *Pst*-inoculation assay showed that the tomato plants treated with the crushed spores did not acquire disease resistance (supplementary Fig. S3), corroborating that the resistance was due to colonization by *G. margarita* and probably not to the spore-associated bacteria or endobacteria alone.

### Defense-related signaling in the G. margarita-colonized tomato plants

**Figure 2:**
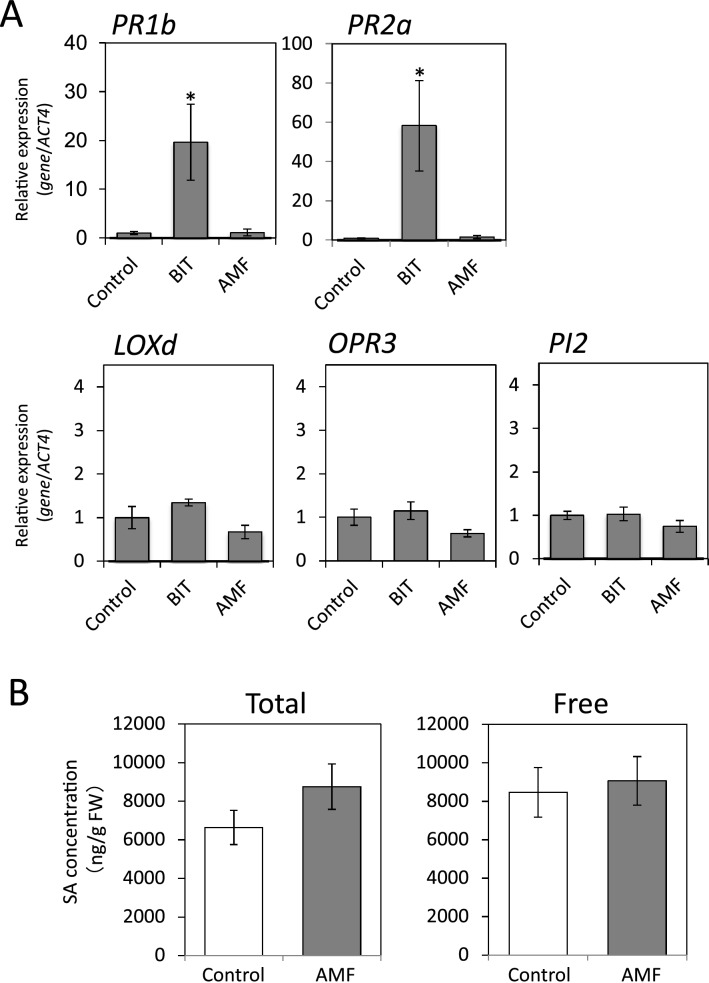
(**A**) Expression of defense-related genes in *G. margarita*-colonized tomato plants. Terminal leaflets of the 4th compound leaves were collected 14 days after the inoculation with *G. margarita* (AMF). SAR was induced by treatment with BIT (5 mg/pot) by soil drenching for 5 days. Real time PCR analysis was performed to evaluate the expression of SAR marker genes (*PR1b* and *PR2a*) and JA-related genes (*Loxd*, *OPR3* and *PI2*). Transcript levels were normalized to the expression of *ACT4* measured in the same samples. The means and SEs were calculated from 4 independent samples, each taken from a single plant. Asterisk indicates statistically significant difference between data of the control and BIT-treated plants (two-sided t-test, *p* < 0.05). The experiment was repeated three times with similar results. (**B**) Salicylic acid levels in *G. margarita*-colonized plants. Terminal and its neighboring leaflets of 4th compound leaves were harvested at 14 days after inoculation with *G. margarita* (25 spores/pot) (AMF). The levels of free and total SA (free SA + SA-glucoside) were quantified by HPLC. Values presented are the means ± SE from 6 samples, each prepared from a single plant. The experiment was repeated three times with similar results.

To determine the physiological changes in *G. margarita*-induced disease resistance in tomato plants, we examined whether SAR was induced by *G. margarita* colonization. Transcript levels of SA-responsive genes, *PR1b* and *PR2a*, were strongly increased by the SAR inducer BIT; however, they were not influenced by *G. margarita* colonization (Fig. 2A). Endogenous SA accumulation in leaves was examined 14 days after inoculation with *G. margarita*. The levels of free SA and total SA (free SA + SA-glucoside) in *G. margarita*-colonized plants were not significantly different from those in the water-treated control plants (Fig. 2B), indicating that *G. margarita* colonization had no effect on SA accumulation in tomato plants. Thus, *G. margarita* colonization did not activate SA-mediated defense signaling in tomato plants.

Another type of systemically induced disease resistance is induced via the JA-mediated signaling pathway, which is activated by wounding and attacks by necrotrophic pathogens and insects. To determine whether JA-mediated signal transduction was activated by *G. margarita* colonization in tomato plants, the expression of JA-related genes was analyzed. Mycorrhizal colonization did not influence the expression of the JA biosynthesis-related genes *LOXd* (encoding lipoxygenase), *OPR3* (encoding 12-oxophytodienoate reductase 3), or the JA-responsive gene *PI2* (encoding protease inhibitor 2) (Fig. 2A), nor did the treatment with BIT. These results indicated that the JA-mediated defense signaling was not activated by *G. margarita* colonization in tomato plants.

### Accelerated responses to pathogen infection by G. margarita colonization

**Figure 3:**
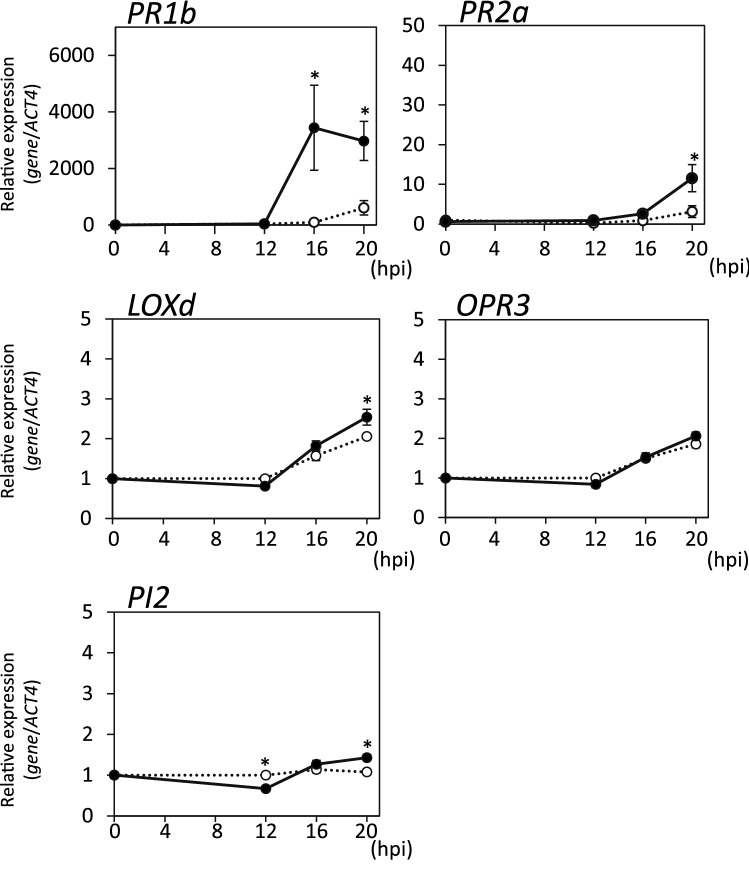
Expression of defense-related genes after infection with the virulent pathogen. The *G. margarita*-colonized (14 days after *G. margarita* inoculation) and the water-treated control tomato plants were inoculated with *Pst*. Leaf disks were taken from the *Pst*-infiltrated part of the leaflets at the indicated time points (0, 12, 16, 20 h post inoculation (hpi)) and used for gene expression analyses of SA-related genes (*PR1b* and *PR2a*) and JA-related genes (*LOXd, OPR3* and *PI2*). Transcript levels were normalized to the expression of *ACT4* measured in the same samples. The means and SEs were calculated from 4 independent samples, each prepared from a single plant. Open circle, water-treated control plant; closed circle, *G. margarita*-colonized plants. Asterisks indicate statistically significant difference between data of the water-treated control and *G. margarita*-colonized plants (two-sided t-test, *p* < 0.05). The experiment was repeated three times with similar results.

To determine whether *G. margarita* colonization had any effects on the responses to pathogens in tomato plants, we examined the expression of defense-related genes after the infection with *Pst*. While the transcript levels of SA-related genes *PR1b* and *PR2a* increased from 20 h after infection with *Pst* in the water-treated control plants, a rapid increase in those transcripts was observed in *G. margarita*-colonized plants (Fig. 3). At 20 h after *Pst* infection, the transcript levels of *PR1b* and *PR2a* in *G. margarita*-colonized plants were approximately six- and four-fold higher, respectively, than those in the water-treated control plants (Fig. 3). These results indicated that activation of the SA-mediated signaling pathway in response to pathogen infection was accelerated in mycorrhizal plants compared to that in the water-treated control plants.

The expression of JA-related genes after infection with *Pst* was not as strong as that of SA-related genes probably because of the suppression by the SA-mediated defense signal that was activated by the biotrophic pathogen. The expression of *LOXd* and *PI2* was enhanced in the *G. margarita*-colonized plants compared to that in the water-treated control plants, whereas this phenomenon was not observed regarding the *OPR3* gene (Fig. 3). Although the induction levels of JA-related gene expression were quite low, these results indicated that the activation of the JA-mediated signaling pathway in response to pathogen infection was accelerated in mycorrhizal plants.

### Effects of mycorrhizal colonization on responses against avirulent bacterial pathogens

**Figure 4:**
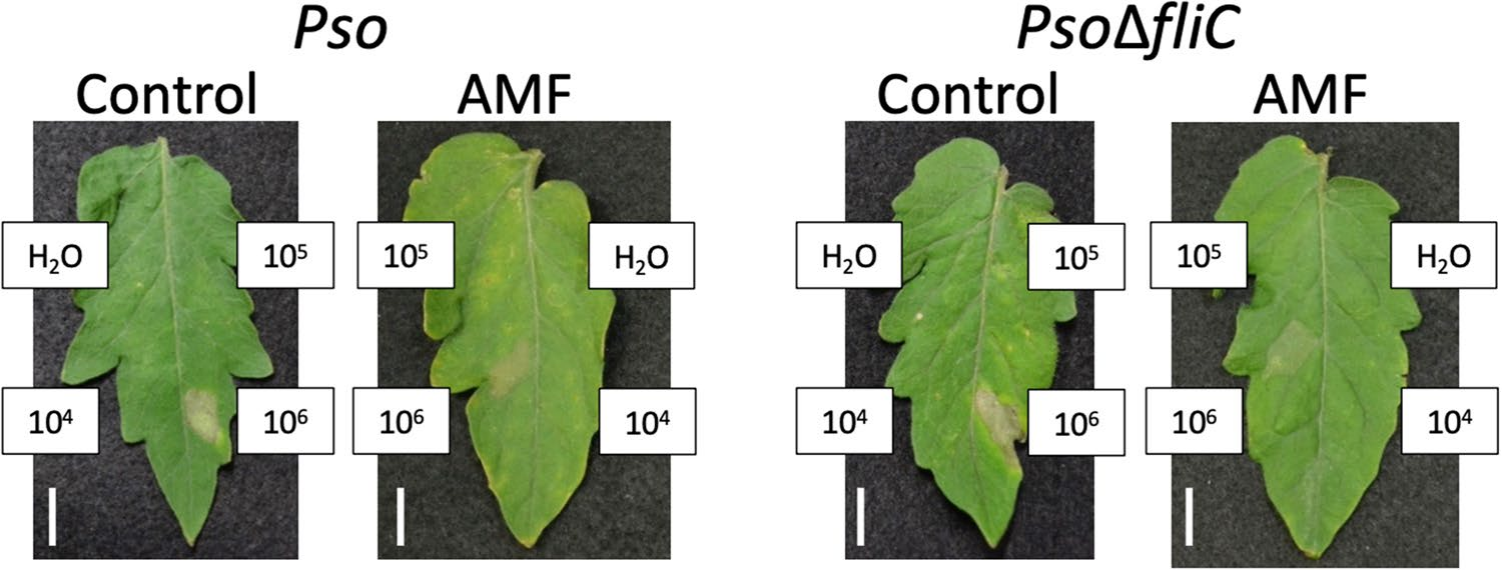
Response of tomato leaves to infection with avirulent pathogens. Tomato plants were inoculated with *G. margarita* (25 spores/pot) for 14 days. Terminal leaflets of the 4th leaves of 3-week-old tomato plants were inoculated with *Pso* or *Pso∆fliC* (1 × 10^4^, 1 × 10^5^, or 1 × 10^6^ CFU/ml) or water (H_2_O). Each experiment was done with more than 4 plants. Photographs were taken 30 h after inoculation. The experiment was repeated twice with similar results. Control, water-treated plants; AMF, *G. margarita*-colonized plants.

Analysis using a virulent pathogen*, Pst*, indicated that *G. margarita*-colonized tomato plants were in the primed state. Furthermore, our results indicated that the priming effects of MIR were provoked not only by fungal but also bacterial pathogens. Despite microorganisms other than virulent pathogens having more opportunities to challenge plants, it is still poorly understood how primed plants respond to avirulent pathogens. To determine how MIR responded to avirulent pathogens, we examined the defense responses of *G. margarita*-colonized tomato plants against an incompatible bacterial strain, *Pseudomonas syringae* pv. *oryzae* (*Pso*). We also used a flagellin-deficient mutant (*Pso∆fliC*) to assess the influence of flagellin as an elicitor in defense response. First, the defense response in the incompatible interaction was examined by infecting a tomato leaflet with different concentrations of these pathogens. In both *G. margarita*-colonized and the control plants, the leaf tissue infected with *Pso* at a concentration of 1 × 10^6^ CFU/mL exhibited a hypersensitive reaction (HR) involving cell death at 18 h after infection; however, lower concentrations of 1 × 10^4^ and 1 × 10^5^ CFU/mL did not cause HRs (Fig. 4). Similar results were obtained by infection with the *Pso∆fliC* mutant, suggesting that the tomato HR against *Pso* was caused by factors other than flagellin (Fig. 4).

**Figure 5:**
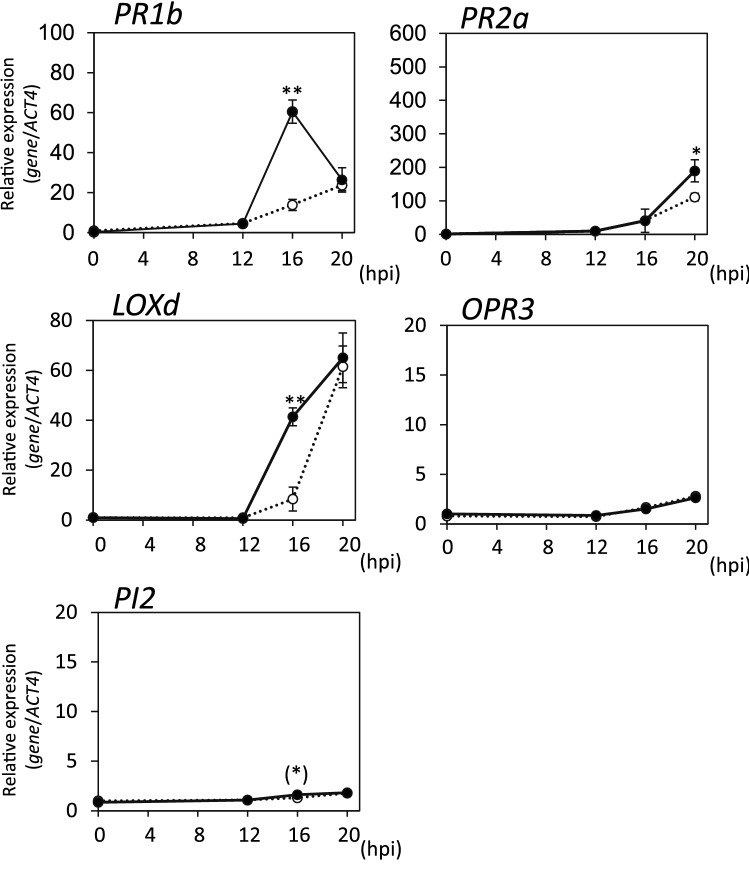
Expression of defense-related genes after infection with avirulent pathogen *Pso.* The *G. margarita*-colonized (14 days after *G. margarita* inoculation) and the water-treated control tomato plants were inoculated with *Pso*. Leaf disks were ﻿taken from the *Pso*-infiltrated part of the leaflets at the indicated time points (0, 12, 16, 20 h post inoculation (hpi)) and used for gene expression analyses of SA-related genes (*PR1b* and *PR2a*) and JA-related genes (*LOXd, OPR3* and *PI2*). Transcript levels were normalized to the expression of *ACT4* measured in the same samples. The means and SEs were calculated from 4 independent samples, each prepared from a single plant. Open circle, water-treated control plant; closed circle, *G. margarita*-colonized plants. Asterisks indicate statistically significant difference between data of the water-treated control and *G. margarita*-colonized plants (two-sided t-test, **, *p* < 0.01; *, *p* < 0.05; (*) in *PI2*, *p* = 0.061). The experiment was repeated three times with similar results.

**Figure 6:**
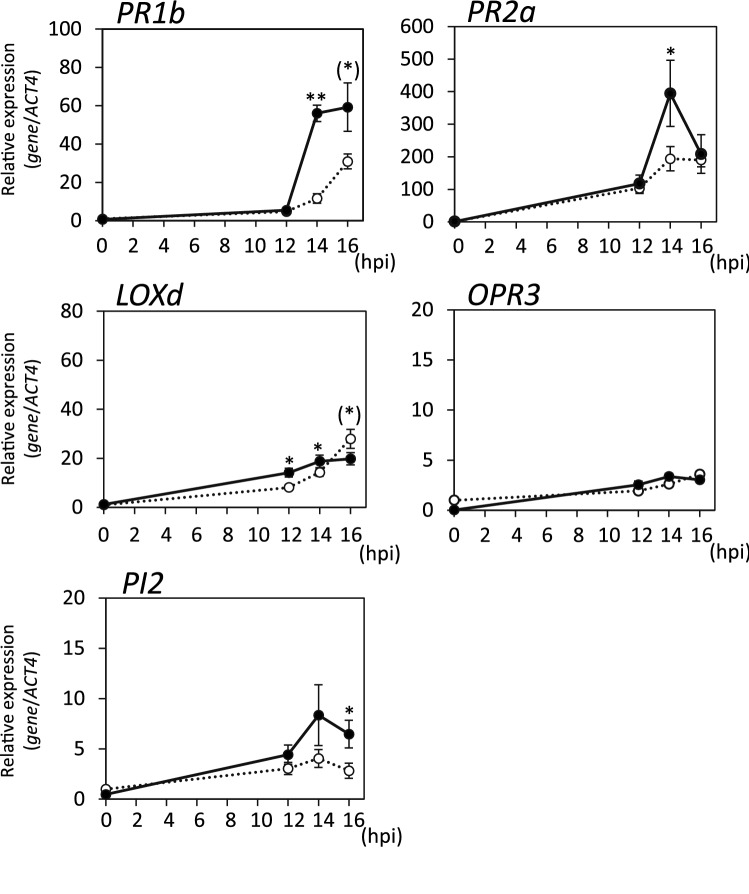
Expression of defense-related genes after infection with avirulent pathogen mutant *Pso∆fliC.* The *G. margarita*-colonized (14 days after *G. margarita* inoculation) and the water-treaterd control tomato plants were inoculated with *Pso∆fliC*. Leaf disks were ﻿taken from the *Pso∆fliC*-infiltrated part of the leaflets at the indicated time points (0, 12, 14, 16 h post inoculation (hpi)) and used for gene expression analyses of SA-related genes (*PR1b* and *PR2a*) and JA-related genes (*LOXd, OPR3* and *PI2*). Transcript levels were normalized to the expression of *ACT4* measured in the same samples. The means and SEs were calculated from 4 independent samples, each prepared from a single plant. Open circle, water-treated control plant; closed circle, *G. margarita*-colonized plants. Asterisks indicate statistically significant difference between data of the water-treated control and *G. margarita*-colonized plants (two-sided t-test; **, *p* < 0.01; *, *p* < 0.05; (*) in *PR1b*, *p* = 0.056; (*) in *Loxd*, *p* = 0.051). The experiment was repeated three times with similar results.

The defense responses against avirulent pathogens were analyzed at the gene expression level by using the same genes used in the *Pst* analysis. The expression patterns of *PR1b* and *PR2a* indicated that the activation of the SA-mediated signaling pathway in response to infection with *Pso* or *Pso∆fliC* was accelerated in *G. margarita*-colonized plants compared to that in the water-treated control plants (Figs. 5 and 6), which was similar to the results with respect to *Pst.* The enhanced expression of *PR1b* by mycorrhizal colonization was much stronger in response to *Pst*, a virulent pathogen (Fig. 3), whereas *PR2a* expression was strongly enhanced in response to avirulent pathogens (Figs. 5 and 6). This difference may be explained by the concentration of inoculants, the compatibility between the host plant and pathogens, and the rapid growth of *Pst* in leaf tissues.

The expression of JA-related genes after the infection with avirulent pathogens was stronger than that with the virulent pathogen *Pst*, probably because of incompatible interactions, such as recognition of avirulent factors and initiation of programmed cell death, which contribute to the activation of JA-mediated signaling pathway (Figs. 3, 5 and 6). Among the JA biosynthesis-related genes tested, *LOXd* expression in response to *Pso* or *Pso∆fliC* was accelerated by *G. margarita* colonization, whereas *OPR3* expression was not influenced (Figs. 5 and 6). Enhancement by mycorrhizal colonization was also observed in the *PI2* expression at 16 h after infection with *Pso∆fliC*, whereas infection with *Pso* had no significant effect (Figs. 5 and 6). These results indicated that the JA-mediated signaling pathway in response to infection with avirulent pathogens, at least with respect to *Pso∆fliC,* was accelerated in *G. margarita*-colonized plants compared to that in the water-treated control plants.

## Discussion

Disease resistance induced by symbiotic soilborne microbes in plants should be an important adaptative strategy to diversified environments, which is also an important mechanism for crop production. To understand the underlying mechanisms at the molecular level, we investigated the defense responses in *G. margarita*-colonized tomato plants to fungal and bacterial pathogens, including incompatible strains. The resistance induced by this mycorrhizal symbiosis revealed protective effects against the fungal pathogen *B. cinerea* and the bacterial pathogen *Pst*. Analyses of gene expression and SA accumulation indicated that the *G. margarita* colonization did not activate the defense signals mediated by SA and JA; however, the activation of these defense signals by infection with *Pst* was enhanced in *G. margarita*-colonized tomato plants, suggesting that the immune system was primed by *G. margarita* colonization. Against an avirulent pathogen*, Pso*, HR induction was not influenced by the priming of tomato plants; however, the SA- and JA-mediated responses to the lower bacterial concentration were enhanced. Thus, the *G. margarita*-primed tomato plants exhibited accelerated induction of defense signaling upon infection with a virulent strain, *Pst*, and with an avirulent strain, *Pso*. Both *Pst* and *Pso* had relatively strong effects on SA- and JA-signaling pathways in tomato, respectively.

*G. margarita* colonization enhanced disease resistance in tomato against necrotrophic pathogen *B. cinerea*, as well as the colonization with *F. mosseae* or *R. irregularis*^13,14,20^. The mechanisms of MIR have been investigated in many plant-mycorrhiza interactions. The tomato-*F*. *mosseae* interaction exhibited a protective effect against *B. cinerea* infection, which was likely related to the lower ABA levels in the mycorrhizal plants compared to those in the control^14^. In rice-*R. irregularis* interaction, defense regulatory genes are activated as a response to *Magnaporthe oryzae* infection^12^. The requirement of JA-mediated defense signaling for MIR was demonstrated in the resistance against *Altenaria solani by* interaction between the JA-biosynthesis-deficient tomato mutant *spr2* and *F. mosseae*^15^.

Since gene expression analyses indicated that *G. margarita* colonization did not activate either SA- or JA-mediated defense signaling (Fig. 2), the disease resistance induced by *G. margarita* colonization was not SAR and the JA-mediated disease resistance but was due to priming of the plant defense system. Gene expression patterns after infection by *Pst* suggested that *G. margarita*-colonized plants were primed and able to respond more rapidly and strongly when challenged by a virulent bacterial pathogen compared to that in the water-treated control plants (Fig. 3). SAR accompanied by the expression of SA-related genes is effective against *Pst* in tomato^32^. Hence, the accelerated activation of the SA-mediated signaling pathway in response to infection presumably plays an important role in the disease resistance against *Pst* in *G. margarita*-colonized plants. Priming was previously reported as a type of resistance mechanism in plants interacting with arbuscular mycorrhizal (AM) fungi^30^, nonpathogenic bacteria^11,33^, and some chemicals^34,35^. Some of these priming mechanisms have been analyzed using bacterial pathogens, whereas the priming mechanisms of MIR have been analyzed only using fungal pathogens. Thus, this is the first demonstration of a priming response against a bacterial pathogen in MIR.

Examining both analyses with virulent and avirulent bacterial pathogens, tomato plants primed by *G. margarita* colonization were found to respond more rapidly and strongly to both types of pathogens, and in which activation of both SA- and JA-mediated signaling pathways could be enhanced (Figs. 3, 5 and 6). The priming effects on each of these hormonal signal transductions differed according to the type of infecting pathogens, which is partly due to the antagonistic crosstalk between SA- and JA-mediated signaling pathways.

In analyses of defense response against avirulent pathogens, infection with *Pso* at the bacterial concentration as *Pst* (1 × 10^5^ CFU/mL) provided unstable and unreliable data on gene expression (data not shown). This result was probably due to either—or both— intense response to the avirulent factors or the initiation of cell death events at an imperceptible rate. To precisely characterize cellular defense responses in mycorrhizal plants by avoiding these unfavorable conditions, we searched for the appropriate bacterial concentration for infection and sampling time points for gene expression analysis. We found that infection with *Pso* at a concentration of 1 × 10^4^ CFU/mL resulted in a similar time course of gene expression patterns to those observed in the case of *Pst*.

Some plant-mycorrhiza interactions activate JA-related defense genes before the pathogen infection^14,15^, whereas tomato-*G. margarita* interaction, under the experimental conditions in this study, did not activate major defense genes, as presented here (Fig. 2). This difference is probably owing to the amount of *G. margarita* inoculum. Previous studies used more than 1,000 spores or propagules as the mycorrhiza inoculum per tomato plant and analyzed the primed plants more than 4 weeks after the mycorrhiza inoculation^14,15^. In this study, we analyzed the primed plant 2 weeks after treating plants with 25 spores of *G. margarita*. Nevertheless, these results indicated that the low colonization rate in our experiment was sufficient to analyze MIR, suggesting that even limited mycorrhizal colonization had the potential to promote systemic signaling to induce MIR. MIR is generally thought to be effective against necrotrophs but not against biotrophs^30^. Recently, it was proposed that SA- and JA-mediated defense signaling pathways were activated in the early and late stages, respectively, of mycorrhizal colonization^36^. In contrast, our study indicated that MIR induction without the activation of SA- and JA-mediated defense was effective against both necrotrophic and biotrophic pathogens. These results suggested that the effectiveness of MIR on necrotrophs or biotrophs was likely dependent on the SA-JA antagonistic crosstalk, although this is not the case for all MIRs*.*

Many plant-mycorrhizal interactions and the involvement of JA in the establishment of MIR, including colonization and defense response to pathogens, have been investigated^15,30^; however, the signal transduction for resistance induction from colonization area to foliar tissues remains to be clarified. As different mycorrhizal symbiosis interactions probably induce different types of MIR, the use of the same bacterial pathogen will enable us to evaluate and compare the mechanisms of disease resistance induced by these MIRs. In this study, we selected the model bacterial pathogen *Pst*, which has been used in *Arabidopsis* research^37^, and used it for the evaluation of the induced resistance in tomato^11,32^. From the phytopathological point of view, a model pathogen provides valuable information to analyze defense mechanisms with respect to MIRs. Since many *Pst* mutants have already been identified and used to investigate the molecular mechanism of plant-pathogen interaction in *Arabidopsis* and also in tomato and tobacco plants^38,39^, they could be a potent tool to clarify the molecular mechanism of MIR in tomato.

In this study, to analyze the interaction between the primed plants and avirulent pathogens, we used the incompatible *Pseudomonas* strain, *Pso*. Gene expression analysis indicated that the primed plants responded rapidly and strongly to this avirulent pathogen, although the *Pso* infection induced HR in both of the primed and control tomato plants (Figs. 4, 5 and 6). Most bacterial infections in nature are assumed to be caused by a low pathogen concentration—too low to induce HR in 24 h, as in the experimental condition—the priming effect on the avirulent pathogen, as shown here, would play an important role in defense. Enhanced responses to *B. cinerea*, *Pst*, and *Pso* suggested that the primed plants are able to recognize a broad range of microbial factors to protect themselves, in which pathogen-associated molecular pattern (PAMP)-triggered immunity (PTI) and effector-triggered immunity (ETI) should take part^40^. As the deletion of bacterial factors of an incompatible strain would provide further information about the defense response of the primed tomato plants, we analyzed the effects of flagellin-deficiency (Δ*fliC*) on the defense response. The enhancement patterns of gene expression were different between *Pso* and *Pso∆fliC,* although it was difficult to conclude that this was owing to the lack of flagellin. Infection with the *fliC* mutant of *P. syringae* pv. *tabaci* in the non-host *Arabidopsis* caused a reduced HR and increased bacterial growth compared to that in the wild-type strain^41^. In the present study, the deletion of the *fliC* gene from *Pso* had no influence on HR under our experimental conditions. However, both defense response and its enhancement by priming, especially the expression of JA-related genes, after infection with this mutant were observed earlier than those with the wild-type strain. This result was probably due to the lack of recognition and response to flagellin or the lack of bacterial motility. Further analyses with other mutants, e.g., defective in *hrcC* and other *hrp* genes, would provide more information about the enhancement mechanism of defense responses in primed tomato plants.

This study demonstrated a fascinating potential of MIR that is effective to both fungal and bacterial pathogens simultaneously even with the low colonization rate. This indicates that symbiotic interaction primes some of the immune systems of host plants. Since a long period-colonization, such as for 2–3 months, activated JA-mediated defense signaling and was effective to fungal pathogens as shown in the tomato-*F. mosseae* interaction^14,15^, the physiological state of primed plants may vary according to the mycorrhizal colonization rates. Thus, analyzing the resistance against bacterial pathogens in other plant-mycorrhiza interactions would reveal the complex regulation mechanism of defense signals in MIR.

## Methods

### Preparation of gigaspora margarita spores

The inoculum of *Gigaspora margarita* Becker & Hall MAFF520054 was propagated using onion (*Allium cepa* L.) cultured in a sterilized soil mixture of a horticultural medium (Kureha Chemical Co., Japan), sand (KOMERI Co., Ltd., Japan), and a humus-rich Andosol (KOMERI Co., Ltd., Japan) (1:5:4 [v/v/v]). Calcium carbonate (CaCO_3_) (1 g/L) was added to maintain a pH of approximately 6. The onion-*G. margarita* co-culture was performed for three months and then air dried for a month. The potting medium containing root debris was maintained for more than a month at 4 °C. Spores were collected from the stocked soil samples using wet sieving followed by picking up using a glass Pasteur pipette (IWAKI, Japan)^42^. The operations were performed under sterile condition.

### Construction of flagellin-deficient mutant of Pso

A flagella-deficient mutant of *Pso* was produced using a homologous recombination method to delete the *fliC* gene encoding flagellin protein. The DNA fragment including the *fliC* gene and adjacent regions of both sides of *fliC* (ca. 1.3 kb and 0.8 kb) were amplified by PCR with KOD-plus and a set of primers tgacttgctttaacctgccaagcg and ggttgccttgaccactgcttcatt, followed by the insertion into pCR-Blunt II-TOPO (Thermo Fisher Scientific, Waltham, MA, USA). The *fliC* gene region was removed by digestion at the *Ssp*I and *Sca* I sites, located outside of the 5’-end and 3’-end of the *fliC* gene, respectively, followed by blunt-end self-ligation. The modified DNA fragment (ca. 2.1 kb) containing only the adjacent regions of *fliC* was transferred from the pCR-Blunt II-TOPO to the mobilizable cloning vector, pK18*mobsacB*^43^*,* by utilizing *Xba*I and *Bam*HI sites in the multi-cloning sites of these vectors. The resulting plasmid pMCPso was introduced into *E. coli* S17-1 by electrotransformation and then transferred to *Pso* by bacterial conjugation. The nalidixic acid-resistant and kanamycin-sensitive *Pso* colonies were selected as the *fliC*-deficient mutant, followed by PCR analysis to confirm the deletion.

### Plant growth condition and mycorrhizal colonization

Tomatoes (*Solanum lycopersicum* L. cv. *Momotaro*, Takii & Co., Ltd, Japan) were sown and grown in sterilized soil (Raising seedling soil, Takii & Co., Ltd, Japan) in plastic pots (5 cm × 5 cm × 5 cm) in a growth chamber (16:8 h L:D, 25 °C, 60% RH). One-week-old tomato seedlings were inoculated with mycorrhizal spores (25 spores per plant) by placing spores with a micropipette at 4 points (3-cm-deep) in the soil, 1.5 cm around the seedling, and returned to the growth chamber. Crushed spores (25 spores in 0.1 mL sterilized water) were prepared by crushing using a pestle with a small amount of fine-ground sea sand in a 1.5 mL plastic tube. To induce SAR, plants were treated with BIT (5 mg/pot) using the soil-drenching method 5 days before pathogen inoculation.

### Pathogen inoculation assays

*G. margarita*-colonized (14 days after inoculation) and the water-treated plants were used for pathogen inoculation assay. *Pst* was cultured in nutrient broth containing rifampicin (100 µg/mL) at 30 °C for 24 h. Bacterial suspension (1 × 10^3^ CFU /mL) prepared using 10 mM MgCl_2_ were infiltrated into the terminal and its neighboring leaflets of the 4th compound leaves using a 1-mL syringe without a needle. Leaf disks (4-mm diameter) were taken from the infiltrated part of the leaflet 2 days after inoculation. Bacterial cells were extracted by homogenizing the leaf disks (5 disks per sample) in 10 mM MgCl_2_. The number of CFUs was estimated by culturing bacterial cells in nutrient broth agar plates after dilution. For each experiment, more than 4 plants were used and 8 samples were prepared. *Pso* and its mutant *Pso∆fliC* were cultured in nutrient broth at 30 °C for 24 h. Bacterial suspensions (1 × 10^4^, 1 × 10^5^, and 1 × 10^6^ CFU/mL in 10 mM MgCl_2_) were prepared and used for inoculation into leaflets of 4th compound leaves.

### Quantification of mycorrhizal colonization

After 14 and 28 days of inoculation with *G. margarita*, tomato roots were washed gently with water to remove soil debris. Roots were cut into 2-cm segments and used for clearing with 10% KOH, acidifying with acetic acid, and staining with trypan blue^44^. The percentage of root colonization by *G. margarita* was determined by the gridline intersection method using a SZ61 stereo microscope (Olympus, Tokyo, Japan) under bright-field conditions^45^.

### Extraction and analysis of SA

The terminal and its neighboring leaflets of the 4th compound leaves were harvested from the *G. margarita*-colonized (14 days after inoculation) and water-treated plants at the time of pathogen inoculation. Extraction and measurement of free and total SA (free SA + SA-glucoside) was performed as previously described^11^.

### Gene expression analysis

For the gene expression analysis in leaves of the *G. margarita*-colonized (14 days after inoculation) and the water-treated control plants, terminal leaflets of the 4th compound leaves were harvested at the time of pathogen inoculation. These were used for total RNA extraction using Sepasol-RNA I super reagent (Nacalai Tesque, Kyoto, Japan), followed by cDNA synthesis using the PrimeScript RT reagent Kit with gDNA Eraser (Takara Bio, Shiga, Japan). Quantitative RT-PCR was performed using a LightCycler 96 System (Roche, Basel, Switzerland). Thermal cycling conditions consisted of 30 s at 95 °C, 40 cycles of 5 s at 95 °C, and 20 s at 60 °C. The PCR reaction mixture contained 2 µL of tenfold diluted cDNA template, 0.8 µL of primer solution (containing 5 µM each of forward and reverse primers), 6.4 µL Milli Q water, and 10 µL of SYBR Premix Ex Taq II (Takara Bio, Shiga, Japan). Transcript levels were normalized to the expression of *ACT4* measured in the same samples. The gene-specific primer pairs used are as follows: for *PR1b*, forward 5’- CTTGCGGTTCATAACGATGC-3’ and reverse 5’- TAGTTTTGTGCTCGGGATGC-3’; for *PR2a*, forward 5’- TCCCTTTTACTTGTTGGGCTTC-3’, reverse 5’- GGGCATTAAAGACATTTGTTTCTGG-3’; for *LOXd*, forward 5’-ATCTTGATGCTTTCACCGACA-3’, reverse 5’-ACACTGCTTGGTTGCTTTTCTTC -3’;for *OPR3*, forward 5’-TCGTTTAATGAGGACTTTGAGGAAC-3’, reverse 5’-AGGATTAGAGATGAAAAGACGACCA-3’; for *PI2*, forward 5’-ACGAAGAAACCGGCAGTGA-3’, reverse 5’-TTGCCTCCACCGAAAACC-3’; for *ACT4*, forward 5’-TTGACTTGGCAGGACGTGA-3’, reverse 5’-CAGCTGAGGTGGTGAACGAG-3’.

### Analysis of defense responses to pathogen infection

The culture and preparation of bacterial suspensions were performed using a method similar to that used for the pathogen-inoculation assay. The bacterial concentration of *Pst* was 1 × 10^5^ CFU/mL, whereas those of *Pso* and *Pso∆fliC* were 1 × 10^4^ CFU/mL to avoid the quick response leading to HR. Bacterial suspensions were infiltrated into terminal leaflets of the 4th compound leaves of the *G. margarita*-colonized (14 days after inoculation) and control plants, followed by a sampling of leaf tissues from the pathogen-infiltrated parts at several time points after inoculation. These were used for RT-PCR analysis. For each time point, more than 4 plants were used and 6 RNA samples were prepared.

### Plant material collection and use permission

No permission is required for plant material as it was purchased from certified dealer of local area.

### Ethics approval and consent to participate

The study has been conducted without violating any ethical codes of conduct.

## Supplementary Information

Supplementary Information.

## Data Availability

All data generated or analyzed during this study are present in this paper and the supplementary materials.
