# Supplementary material for: Response of tomatoes primed by mycorrhizal colonization to virulent and avirulent bacterial pathogens
Source: Sci Rep. 2022 Mar 18;12:4686. doi: 10.1038/s41598-022-08395-7 (PMC8933586; doi:10.1038/s41598-022-08395-7)
Supplement: Supplementary file 1 — Supplementary Information. [file 41598_2022_8395_MOESM1_ESM.pdf]

Supplementary Information

Response of tomatoes primed by mycorrhizal colonization to virulent and avirulent bacterial pathogens

Moeka Fujita[1#], Miyuki Kusajima[1#], Masatomo Fukagawa[1], Yasuko Okumura[1], Masami Nakajima[2], Kohki Akiyama[3], Tadao Asami[4], Koichi Yoneyama[5], Hisaharu Kato[1], Hideo Nakashita[1]*

[1]Department of Bioscience and Biotechnology, Fukui Prefectural University, Eiheiji, Japan

[2]*Faculty of Agriculture, Ibaraki University, Ami, Japan*

[3]*Graduate School of Life and Environmental Sciences, Osaka Prefecture University, Sakai, Japan*

[4]*Graduate School of Agricultural and Life Sciences, The University of Tokyo, Tokyo, Japan*

[5]*Center for Bioscience Research and Education, Utsunomiya University, Utsunomiya, Japan*

*Corresponding author:

Hideo Nakashita

E-mail: nakashita@fpu.ac.jp

TEL: +81-776-61-6000

FAX: +81-776-61-6011

[#]These authors contributed equally to this work.

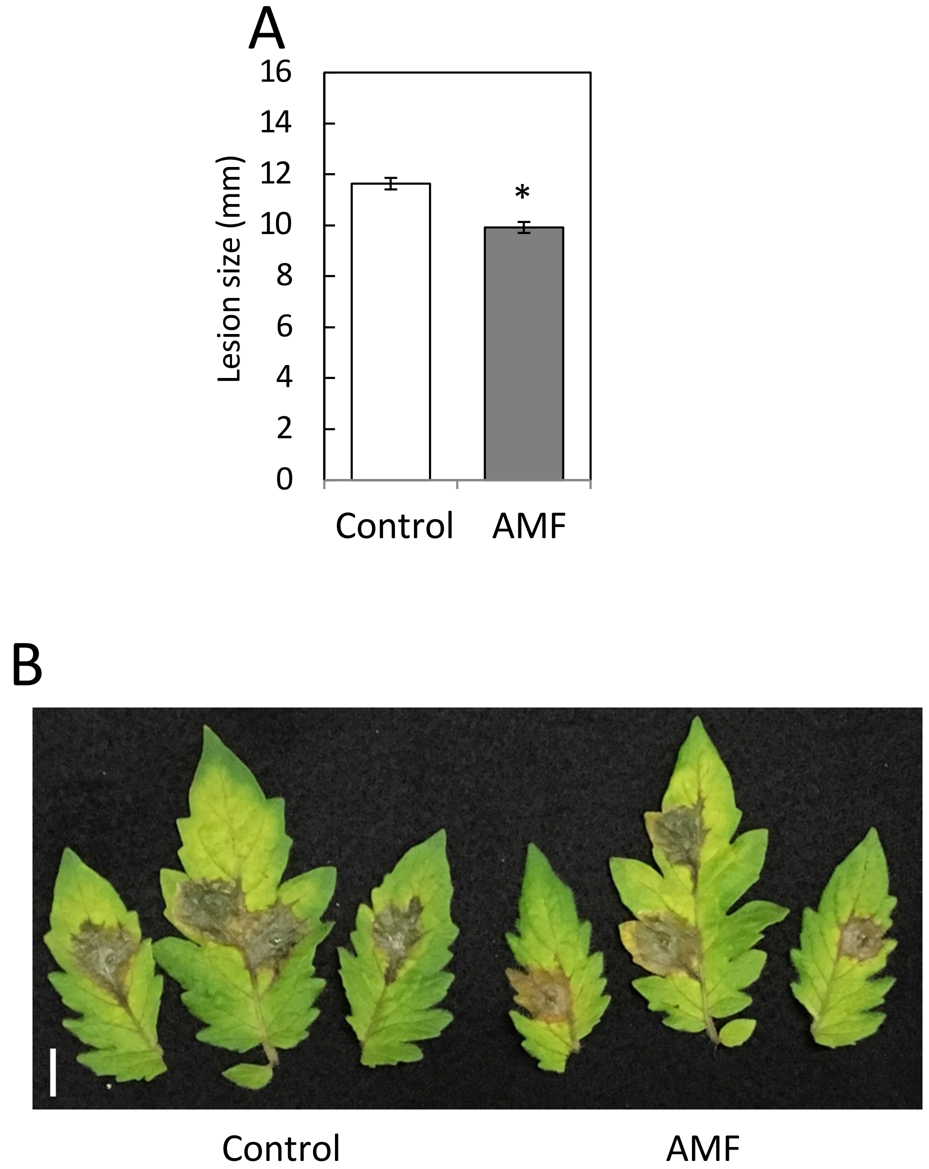

Figure S1. Induction of resistance against tomato gray mold disease by *Gigaspora margarita*.

Tomato plants (3-week-old) were treated with *G. margarita* (25 spores/pot) (AMF) 14 days prior to challenge inoculation with *B. cinerea* strain TV335. *B. cinerea* strain TV335 was cultured in a potato dextrose agar (BD, NJ, USA) plate at 20˚C. Spore formation and inoculation of tomato leaves were performed as previously described [11]. (A) Each experiment contained 30-40 leaflets of 8 plants and the size of spreading lesions were measured 2 days after challenge inoculation. Means ± SE (n=30-40) are presented. Asterisk indicates statistically significant difference between data of the water-treated control and *G. margarita*-colonized groups (two-sided t-test, $p < 0.05$). The experiment was repeated three times with similar results. (B) Photograph of representative disease symptoms taken 2 days after inoculation with *B. cinerea*. Scale bar, 10 mm.

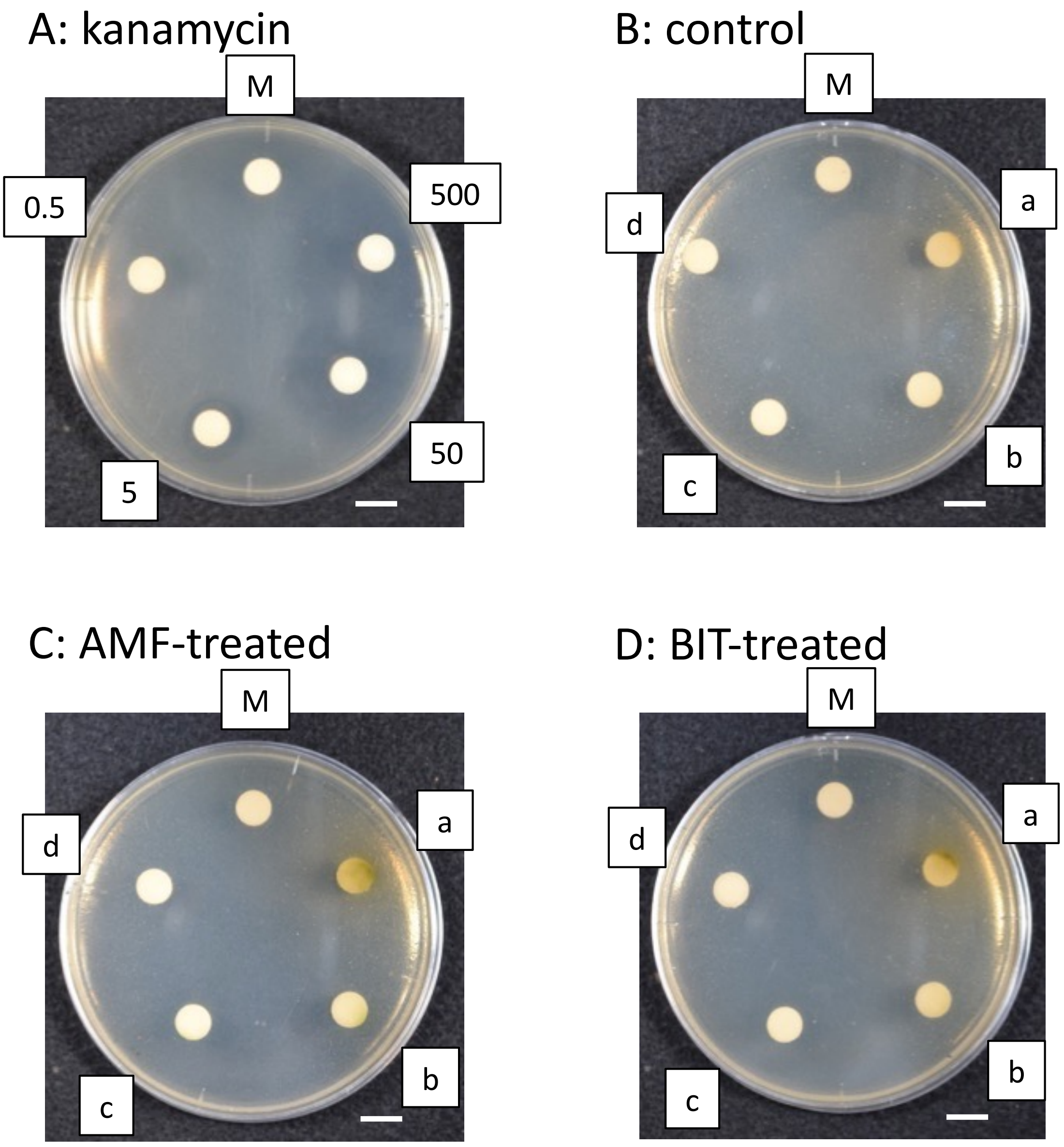

Figure S2. Paper disk assay for antimicrobial activity against *Pseudomonas syringae* pv. *tomato*.

Leaf extracts were prepared by homogenization and centrifugation from leaf samples (100 mg) taken 2 weeks after *G. margarita*-inoculation (AMF) or 5 days after BIT-treatment. Paper disks (6mm, Whatman) containing 10 µl of leaf extract or its diluents were placed on the NB medium plate containing *Pst* (1 x 10^5 CFU/ml) and cultured for 2 days at 28˚C. Scale bar, 10 mm. (A) Kanamaycin (500, 50, 5 , and 0.5 µg/ml) and water (M). (B, C, D) a, leaf extract; b 5-fold dilution; c, 25-fold dilution; d, 125-foud dilution; M, water.

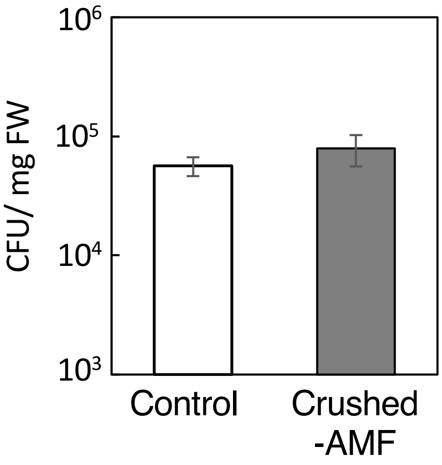

Figure S3. Effect of treatment with crushed-AMF spores on disease resistance in tomato plants.

Plants were treated with crushed spores of *G. margarita* (25 spores/plant) (Crushed-AMF) 14 days prior to challenge inoculation with *Pst* (1 x 10^5 CFU/ml). The growth of *Pst* in tomato leaflet was evaluated 2 days after the inoculation. Each experiment was done with four plants. Values are shown as the means ± SE (n=8) of a single experiment.
